# Annealing Lamp

**Published:** 1865-02

**Authors:** 


### ANNEALING LAMP.

We have for some time been using a small conveniently arranged annealing lamp for dentists' use, for annealing gold before filling. In it the gold is kept hot all the time, just as the operator desires it. It occupies but little space and is very efficient for that for which it was designed. Every dentist should have one upon his case; he will find it valuable, perhaps, for other purposes than annealing gold.

It was invented and constructed by Dr. A. J. Metcalf, of Kalamazoo, Mich. They are for sale at the Dental Depots. The price varies according to the style of lamp.

